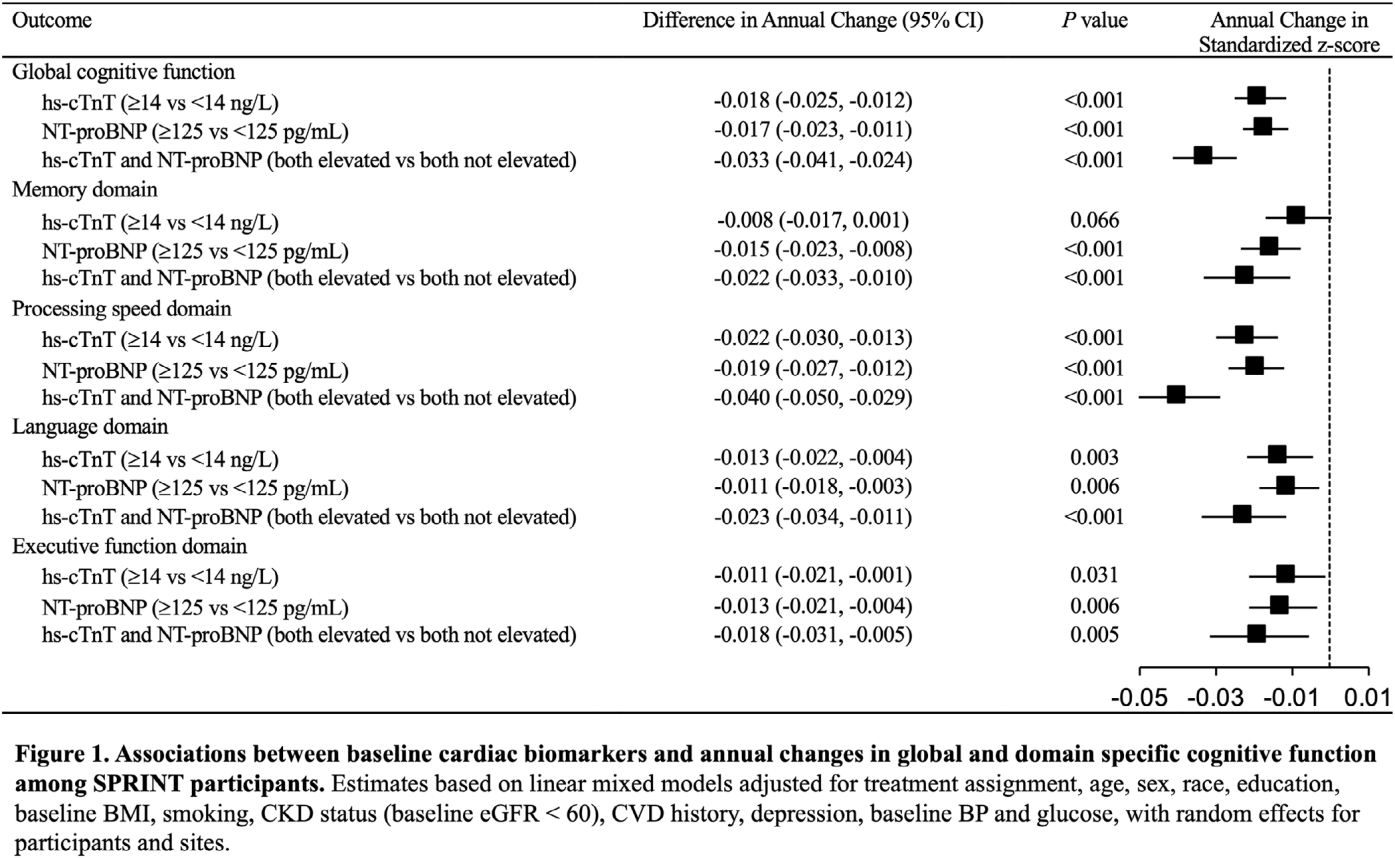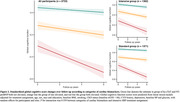# Cardiac Biomarkers, Subclinical Brain Vascular Changes, and Cognitive Decline: Post Hoc Analysis of the SPRINT Trial

**DOI:** 10.1002/alz.087991

**Published:** 2025-01-09

**Authors:** Wenxin Zhang, Simon B. Ascher, Sudipto Dolui, Ilya M. Nasrallah, Yuan Lu, Julia Neitzel, Estefanía Toledo, Lidia Glodzik, Timothy M. Hughes, Jarett D Berry, Yuan Ma

**Affiliations:** ^1^ Harvard T.H. Chan School of Public Health, Boston, MA USA; ^2^ San Francisco Veterans Affairs Health Care System and University of California San Francisco, San Francisco, CA USA; ^3^ Department of Radiology, University of Pennsylvania, Philadelphia, PA USA; ^4^ University of Pennsylvania, Philadelphia, PA USA; ^5^ Yale School of Medicine, New Haven, CT USA; ^6^ Erasmus University Medical Center, Rotterdam Netherlands; ^7^ Universidad de Navarra, Pamplona Spain; ^8^ Weill Cornell Medicine, New York, NY USA; ^9^ Wake Forest University School of Medicine, Winston‐Salem, NC USA; ^10^ University of Texas Southwestern Medical School, Dallas, TX USA

## Abstract

**Background:**

Subclinical cardiovascular disease (CVD), assessed by high‐sensitivity cardiac troponin T (hs‐cTnT) and N‐terminal pro‐B‐type natriuretic peptide (NT‐proBNP), is linked to cognitive decline, but the associations in hypertensive adults and the underlying brain pathologies remain unclear. It is also undetermined whether an intensive blood pressure treatment compared to a standard treatment may slow down cognitive decline associated with subclinical CVD.

**Method:**

We conducted a post hoc analysis of the Systolic Blood Pressure Intervention Trial, where older adults with hypertension were randomized to an intensive treatment (systolic blood pressure (SBP) target of < 120 mm Hg) or standard treatment (< 140 mm Hg). Serum hs‐cTnT and NT‐proBNP concentrations were measured at baseline, with elevated levels defined as ≥ 14 ng/L for hs‐cTnT and ≥ 125 pg/mL for NT‐proBNP. Global and domain‐specific (memory, processing speed, language, and executive function) cognitive function was assessed using a battery of neuropsychological tests at baseline and during the follow‐up (year 2, year 4, and year 6) in 2733 participants with biomarker measurements. White matter lesions, cerebral blood flow, and brain tissue volume were assessed by brain MRI at baseline and 4 years of follow‐up in a subset of 639 participants.

**Result:**

Elevated levels of both hs‐cTnT and NT‐proBNP were associated with accelerated decline in cognitive function across all four domains after adjusting for potential confounding factors. The group with elevated levels of both cardiac biomarkers showed the fastest decline, with a greater annual decline rate of 0.033 (95% CI, 0.024 to 0.041) in standardized z‐score of global cognitive function compared to the group with normal hs‐cTnT and NT‐proBNP. Elevated levels of both cardiac biomarkers were also associated with a faster progression in white matter lesions, but not with changes in total brain tissue volume and cerebral blood flow. Intensive SBP treatment did not attenuate these associations compared to standard treatment (all *P* for interaction > 0.05).

**Conclusion:**

Elevated hs‐cTnT and NT‐proBNP levels were associated with accelerated cognitive decline and progression of white matter lesions regardless of SBP treatment assignment. Subclinical CVD may contribute to the progression of white matter lesions and thus leads to accelerated cognitive decline.